# Prevalence, types, and risk factors of functional gastrointestinal diseases in Hainan Province, China

**DOI:** 10.1038/s41598-024-55363-4

**Published:** 2024-02-24

**Authors:** Chen Chen, Da-ya Zhang, Shiju Chen, Shimei Huang, Fan Zeng, Da Li, Yan-ting Lv, Xiaohong Xiang, Run-xiang Chen, Xiaodong Zhang, Fengjiao Mao, Xianfeng Huang, Jun Wang, Feihu Bai

**Affiliations:** 1 Graduate School, Hainan Medical University, Haikou, 571199 China; 2 Department of Pediatrics Affiliated Chifeng Clinical College, Inner Mongolia Medical University, Chifeng, 010110 China; 3 Department of Gastroenterology, The 986 Hospital of Xijing Hospital, Air Force Military Medical University, Xi’an, 710054 China; 4 Department of Gastroenterology, The Second Affiliated Hospital of Hainan Medical University, Yehai Avenue, #368, Longhua District, Haikou, 570216 Hainan China; 5 The Gastroenterology Clinical Medical Center of Hainan Province, Haikou, 570216 China

**Keywords:** Functional gastrointestinal diseases, Prevalence, Risk factors, Hainan Province, China, Diseases, Gastroenterology, Medical research, Risk factors, Signs and symptoms

## Abstract

To investigate the prevalence, types, and risk factors of functional gastrointestinal diseases (FGIDs) in Hainan Province, China, in order to provide insights for future prevention and treatment strategies. A questionnaire survey was conducted from July 2022 to May 2023, using stratified sampling to sample local residents in five cities (20 townships) in Hainan Province. Out of 2057 local residents surveyed, 659 individuals (32.0%) reported experiencing at least one FGID. The most prevalent FGIDs were functional dyspepsia (FD) (10.7%), functional constipation (FC) (9.3%), irritable bowel syndrome (IBS) (6.8%), functional bloating (2.2%), belching disorder (2.2%), functional diarrhea (FDr) (1.5%), functional heartburn (1.5%), and fecal incontinence (0.98%). The study revealed significant associations between FGIDs and factors such as age, sleep quality, anxiety, smoking, alcohol consumption, and the consumption of pickled food (*P* < 0.05). Older age, poor sleep quality, anxiety, and the consumption of pickled food were identified as independent risk factors for the prevalence of FGIDs (*P* < 0.05). In Hainan Province, the overall prevalence of FGIDs was found to be 32.0%, with higher prevalences of FC and FD. Older age, poor sleep quality, anxiety, and the consumption of pickled food were identified as risk factors for FGIDs.

## Introduction

Functional gastrointestinal disorders (FGIDs), now referred to as disorders of gut–brain interaction (DGBI), are a group of digestive disorders that are prevalent worldwide. They include functional constipation (FC), functional diarrhea (FDr), irritable bowel syndrome (IBS), functional dyspepsia (FD), and others^1^. These disorders are characterized by recurrent gastrointestinal symptoms without any organic pathology, with a global prevalence of up to 40% and a prevalence of up to 34.4% in China^1^. In 2016, the diagnostic guidelines for IBS were revised in the Rome IV criteria, resulting in more stringent diagnostic criteria. As a result, the prevalence of IBS decreased significantly with the new criteria that require a higher frequency of episodes^2^. It is important to note that the prevalence rates may vary depending on the diagnostic criteria used. The pathogenesis of FGIDs is believed to be associated with impaired gut–brain interactions^3^. The microbial–gut–brain axis (MGBA) refers to the bidirectional regulatory interplay between gut microbes and the brain. Gut microbes can influence gastrointestinal tract function through various pathways^4^. Additionally, abnormal digestive tract dynamics, infections, psychosomatic factors, and dietary habits have also been linked to FGIDs^5–9^. The incidence of FGIDs may differ across regions due to variations in dietary and lifestyle habits. Conducting epidemiological surveys can provide a better understanding of the incidence of FGIDs as well as their unique characteristics. However, there is currently a lack of large-scale epidemiological surveys in Hainan Province, China. Hainan Province is a tropical island surrounded by the sea on all sides. The region is relatively economically and medically underdeveloped, with limited scientific research. Moreover, there are no clinical centers focused on digestive diseases in Hainan Province until 2022. As a result, there is no organization that can effectively collaborate with community hospitals and neighborhood committees to conduct large-scale epidemiological surveys in the region.

## Results

### Prevalence

A total of 2057 valid questionnaires were collected from residents in Hainan Province, consisting of 586 males and 1471 females. The participants had a mean age of 46.86 ± 0.33 years. According to the Rome IV diagnostic criteria, it was found that 659 individuals out of the total sample had at least one FGID, resulting in a prevalence rate of 32.0%. Specifically, the prevalence of FGIDs in males was 30.9%, while it was slightly higher in females at 32.5%. Among the various types of FGIDs, functional dyspepsia and functional constipation had the highest prevalence rates at 10.7% (220/2057) and 9.3% (191/2057), respectively. The remaining FGIDs, including irritable bowel syndrome (IBS), functional bloating, belching disorder, functional diarrhea, functional heartburn, and fecal incontinence, had prevalence rates of 6.8% (140/2057), 2.2% (47/2057), 2.2% (46/2057), 1.5% (30/2057), 1.5% (32/2057), and 0.98% (20/2057), respectively (Table 1). This study was carried out in multiple communities across five cities in Hainan Province. Specifically, within Dongfang City, 154 out of 408 individuals reported having an FGID, resulting in a prevalence rate of 37.7%. Haikou City had a prevalence rate of 25.4%, as 89 out of 350 participants were affected. In Wuzhishan City, the prevalence rate was 35.0%, with 173 out of 494 individuals reporting FGIDs. Qionghai City had a prevalence rate of 28.6%, as 79 out of 276 participants had FGIDs. Similarly, in Sanya City, the prevalence rate was 31.0%, with 164 out of 529 individuals being affected (Figs. S1 and S2).

**Table 1:** Prevalence of FGIDs.

	Amount	Prevalence (%)
	(N = 2057)	
FGIDs	659	32.0
Functional dyspepsia (FD)	220	10.7
Functional Constipation (FC)	191	9.3
Irritable Bowel Syndrome (IBS)	140	6.8
Belching	46	2.2
Functional Bloating	47	2.2
Functional diarrhea (FDr)	30	1.5
Functional Heartburn	32	1.5
Fecal incontinence	20	0.98
Equal to or more than 2	65	3.2

### Analysis of risk factors for FGIDs

In the analysis of this study, various factors were examined to determine their association with the prevalence of FGIDs. The results indicated that age, sleep quality, anxiety, smoking, alcohol consumption, and consumption of pickled food were significantly correlated with the prevalence of FGIDs (*P* < 0.05) (Table 2). On the other hand, gender, mental illness, education level, exercise time, and betel nut consumption did not show any correlation with the disease (Table 2). Furthermore, the analysis revealed that old age, poor sleep quality, anxiety, and consumption of preserved foods were identified as independent risk factors for FGIDs (*P* < 0.05) (Table 3). When looking at specific types of FGIDs, it was found that poorer sleep quality, smoking, and alcohol consumption were independent risk factors for functional dyspepsia (*P* < 0.05) (Tables S1, S2). Additionally, less exercise time and eating pickled food were identified as independent risk factors for functional constipation (*P* < 0.05) (Tables S3, S4). Finally, older age, poor sleep quality, and anxiety were determined to be independent risk factors for irritable bowel syndrome (*P* < 0.05) (Tables S5, S6). In summary, this study provided valuable insights into the risk factors associated with the prevalence of FGIDs. The findings suggest that interventions targeting sleep quality, anxiety, smoking, alcohol consumption, and dietary habits may have a significant impact on managing and preventing these conditions. Further research in this area is needed to validate and expand upon these results.

**Table 2:** Univariate analysis of prevalence of FGIDs.

Indicator	Healthy group	Diseased group	X^2^/t	P value
Age (years)				
18–40	548	214	8.87	< 0.05
41–60	581	299		
> 60	269	146		
Gender				
Male	405	181	0.49	0.48
Female	993	478		
Sleep quality				
Good	536	171	34.33	< 0.05
Average	540	280		
Poor	322	208		
Anxieties				
Hardly	707	261	24.16	< 0.05
Occasionally	476	256		
Often	215	142		
Psychiatric disorders				
No	1362	640	0.16	0.69
Yes	36	19		
Educational level				
Undergraduate and above	396	161	0.17	3.52
Elementary-high school	938	468		
Never attended school	64	30		
Exercise duration/week				
< 1 h	644	330	0.23	2.96
1–4 h	440	189		
> 4 h	314	140		
Smoking				
Not	1191	539	3.88	< 0.05
Yes	207	120		
Drinking alcohol				
Not	1228	555	5.08	< 0.05
Yes	170	104		
Eating pickled foods				
Not	1007	436	7.37	< 0.05
Yes	391	223		
Edible betel nut				
Not	1101	523	0.09	0.75
Yes	297	136

**Table 3:** Multifactorial analysis of the prevalence of FGIDs.

Indicator	Subgroup	P value	OR	95% CI	
				Lowest	Highest
Age (years)	18–40	< 0.05	1.000		
	41–60	< 0.05	1.332	1.068	1.662
	> 60	< 0.05	1.480	1.127	1.945
Sleep quality	Good	< 0.05	1.000		
	Average	< 0.05	1.519	1.206	1.913
	Poor	< 0.05	1.755	1.361	2.264
Anxieties	Hardly	< 0.05	1.000		
	Occasionally	< 0.05	1.392	1.119	1.731
	Often	< 0.05	1.555	1.191	2.030
Smoking	Not	0.064	1.281	0.986	1.663
	Yes				
Drinking alcohol	Not	0.354	1.141	0.863	1.508
	Yes				
Eating pickled foods	Not	< 0.05	1.267	1.029	1.560
	Yes				

## Discussion

The Rome Foundation conducted a comprehensive global survey on FGIDs and obtained responses from a large sample size of 54,127 participants. The survey revealed that at least 40.3% of the respondents met the diagnostic criteria for FGIDs. Notably, China had a slightly lower prevalence of FGIDs at 34.4%, although certain subtypes such as functional constipation (10.6%) and functional dyspepsia (5.9%) were more prevalent in the country^1^. In our study conducted in Hainan Province, we observed a FGID prevalence of 32.0%. These findings are consistent with the observations made by the Rome Foundation and other scholars, which suggest a higher incidence of functional constipation and functional dyspepsia in our country. To the best of our knowledge, our study is the first in the Chinese tropics to apply the Chinese translated version of the Rome IV criteria for FGIDs. Our study will provide a good reference value for all tropical regions in China and around the world. Besides, Our findings may be suggestive of the prevalence of FGIDs in the tropics after the COVID-19 epidemic. COVID-19 pandemic has significantly changed lifestyles and cause stress worldwide. Recognizing the prevalence of GI sequelae associated with COVID-19 has important implications for clinical strategies and choices in patient care, and for giving patients more attention to GI sequelae during follow-up, as well as for the rational allocation of nursing and medical resources.

In our study, the prevalence of FD appeared to be higher compared to previous findings. For instance, a study conducted among tropical island workers in China found a prevalence of FD as high as 12.77%^10^. Another study in Mexico, a tropical and subtropical region, reported a prevalence rate of 7.0% for Uninvestigated Dyspepsia^11^. It is intriguing to note that tropical regions seem to exhibit a higher prevalence of FD. This observation may be attributed to the unique environmental conditions prevalent in these areas, characterized by high temperatures and humidity. Such conditions make the contamination of drinking water and food more likely, potentially leading to intestinal inflammation^12^. Furthermore, a study conducted on mice revealed a significant difference in the composition of intestinal flora, specifically the genus Thickwellia, between mice living in high-temperature environments and those in room temperature conditions^13^. Factors such as intestinal inflammation, alterations in intestinal flora, loss of appetite^14^, and the distinct dietary culture of Hainan may collectively contribute to the development of dyspepsia.

According to the Rome III diagnostic criteria, the prevalence of IBS is approximately 8.4%^15^. However, the Rome IV diagnostic criteria suggest that the prevalence of IBS may be only half of that under the Rome III criteria^1,16,17^, ranging from 4.4 to 4.8%^18^. Interestingly, our study conducted in Hainan found a higher prevalence of IBS, reaching 6.8%. One possible contributing factor is the increased incidence of IBS after COVID-19 infection^19,20^. It is plausible that novel coronavirus infection can induce pathological changes in the gastrointestinal tract, such as dysbiosis, disruption of intestinal barrier function, and inflammation, which may elevate the prevalence of IBS^20,21^. Furthermore, COVID-19 infection has been associated with persistent psychological burdens (e.g., anxiety and depression), sleep disorders^22,23^, and lifestyle changes, all of which can impact gastrointestinal function. These findings shed light on the potential prevalence of FGIDs in tropical regions following the COVID-19 pandemic.

The prevalence of FGIDs varies across different regions of Hainan Province, with Haikou City exhibiting the lowest incidence rate. Haikou, being the capital of Hainan Province, boasts a higher level of economic development compared to other areas. It also benefits from a well-established healthcare system, a major airport, and a harbor and has embraced various external food cultures and lifestyles in its progress. These factors may contribute to the lower prevalence observed in Haikou.

Our study findings indicate that several factors, namely old age, poorer sleep quality, anxiety, and consumption of pickled foods, are independent risk factors for the prevalence of FGIDs. Consistent with previous research^1,24^, our study found a higher prevalence of FGIDs in females (32.5% > 30.9%), although the correlation between gender and the disease was not statistically significant (*P* > 0.05). Previous studies have demonstrated that FGIDs, particularly FC, are associated with old age, poor sleep quality (odds ratio [OR] = 2.14), and psychological distress (OR = 3.16)^25^. In our study, both old age and anxiety were identified as risk factors for FGIDs. The strong association between FGIDs and mental health is well established^26,27^. The underlying mechanisms through which psychological conditions contribute to the prevalence of FGIDs have not been fully elucidated. Some researchers propose that psychological disorders disrupt gastrointestinal function by activating mast cells and their corresponding mediators, which in turn affect the enteric nerves^28,29^. Others suggest that psychological disorders influence the brain-gut axis, alter gut-specific autonomic output signals, and result in visceral hypersensitivity through regulatory mechanisms involving 5-hydroxytryptamine (5-HT)^30^. Consequently, central modulators are frequently used in the clinical management of FGIDs^31,32^. However, in our study, only anxiety showed a significant correlation with the disease, while psychiatric disorders did not exhibit a statistically significant association with FGIDs (*P* < 0.05), which is difficult to explain.

We also investigated the association between FGIDs and other factors, such as sleep quality and smoking. Our study found that both poor sleep quality and smoking were associated with FGIDs, with poor sleep quality being an independent risk factor for the development of FGIDs. Sleep disturbances can disrupt circadian rhythms, leading to abnormal gastrointestinal dynamics, which can manifest as gastrointestinal discomfort, including diarrhea or constipation, as well as disturbances in intestinal flora composition, such as decreased beneficial bacteria and increased harmful bacteria^33^. The mechanism underlying the association between smoking and gastrointestinal symptoms remains unclear. However, previous studies have demonstrated a link between smoking behavior and various digestive disorders, including gastroesophageal reflux disease, esophageal cancer, peptic ulcer, and gastric cancer^34^. Additionally, our independent analysis identified low exercise time as another risk factor for functional constipation, which could be related to alterations in gut microbiota^35^. Exercise has the potential to modulate the gut microbial composition, promoting gastrointestinal health. Specifically, exercise can increase the abundance of bifidobacteria in the intestines, which are known to produce short-chain fatty acids (SCFA) such as butyric and propionic acids and lower colonic pH. The lower pH environment enhances intestinal motility and reduces colonic transit time^36,37^.

Hainan Province, a coastal region with a unique dietary culture, including the consumption of betel nut and pickled seafood^38^, was investigated in this study to explore the factors influencing the prevalence of FGIDs. The findings revealed that betel nut consumption did not show a significant association with FGIDs (*P* > 0.05), while the consumption of pickled food increased the risk of FGIDs (*P* < 0.05 OR = 1.267). This relationship between pickled foods and FGIDs has been rarely studied in previous research. Pickled foods are known to contain high levels of nitrite, and it has been observed that mice ingesting nitrite were more prone to developing intestinal flora disorders compared to those not ingesting nitrite^39^. This suggests that the pathogenesis of FGIDs caused by pickled foods may involve the disruption of intestinal flora, although further research is needed to confirm this hypothesis.

This study has several limitations that should be addressed. Firstly, the survey was conducted in a single region, which may limit the generalizability of the findings. It is possible that there could be geographic variability in the prevalence of the factors studied. Additionally, certain important factors such as *Helicobacter* *pylori* infection, history of novel coronavirus infection, and dietary patterns were not included in the study. Previous research has suggested that *H*. *pylori* infection may be associated with symptoms of functional dyspepsia^40,41^, and novel coronavirus infection is often accompanied by gastrointestinal symptoms^42,43^. Therefore, the exclusion of these effects in our study is unfortunate and may impede a comprehensive understanding of the issue. Furthermore, in this study, respondents did not undergo gastroenteroscopy, but rather, their history of gastrointestinal disorders and previous gastroenteroscopic findings were investigated. This approach may not provide a complete exclusion of organic gastrointestinal pathology. It is important to note that this limitation is common in studies on FGIDs, as it can be challenging to obtain refined gastroenteroscopic data from respondents^1,44,45^. Moreover, many studies, including online surveys, rely on past medical histories to ascertain the presence of organic pathology. According to our survey, healthy and mildly symptomatic individuals expressed reluctance towards undergoing gastroenteroscopy due to discomfort associated with the invasive procedure.

## Conclusion

In Hainan, the prevalence of FGIDs was found to be 32.0%. Among FGIDs, there was a higher prevalence of FC and FD. Factors such as old age, poor sleep quality, anxiety, and consumption of preserved foods were identified as increasing the risk of developing FGIDs. To summarize, adopting healthy sleep patterns, reducing anxiety levels, engaging in regular exercise, quitting alcohol consumption, and avoiding the consumption of pickled foods can help in lowering the prevalence of FGIDs.

## Materials

### Sample size estimation

The sample size estimation method was utilized to determine the number of participants needed for this cross-sectional survey study. Previous epidemiological data from China reported a prevalence of FGIDs (Functional Gastrointestinal Disorders) according to the Roman IV diagnostic criteria at around 34.4%^1^. Therefore, in this study, the sample size calculation was taken p = 0.34, α = 0.05, μα/2 = 1.96, r = 6.2%, δ = r·p. The sample size formula for the estimation of the overall rate is: N = μ2α/2P(1 − P)/δ2 = μ2α/2(1 − P)/r2·P = 1940. Considering a failure rate of 10–20%, We decided to maximize the sample size as much as possible and planned to sample 2300 people.

### Study population and design

Hainan Province, located in the South China Sea, is a tropical island surrounded by the ocean. For the purpose of this study, we have carefully selected five cities in different regions of the province to ensure a representative sample. These cities include Dongfang City in the east, Haikou City in the west, Wuzhishan City in the south, Qionghai City in the north, and Sanya City in the center. According to the results of the seventh national census^46^, the total population of Hainan Province is approximately 10 million people. Among these cities, Haikou and Sanya have the highest population, each exceeding one million residents. Qionghai City has a population between 500,000 and 1 million, while Dongfang City has a population between 200,000 and 500,000. Wuzhishan City, on the other hand, has a population of less than 200,000. In our previous epidemiological surveys on *Helicobacter* *pylori* infection rates in Hainan Province^47^, published in international medical journals, we have gained significant experience in this field. Building on this expertise, we have selected these five regions as they are representative in terms of ethnicity, economic development, dietary habits, and geographical location.

The study took place between July 2022 and May 2023, with each municipality selecting 4 communes and each commune selecting 4 communities to participate in the survey. Prior to the study, the necessary staffing and publicity preparations were carried out. The study was conducted every 2 months, simultaneously in 4 townships of a city, for a duration of 4 days by gastroenterologists from our hospital. Local community hospitals served as the locations for the survey, where offline one-on-one consultations and completion of questionnaires were the main focus. In addition, a small number of young people collected questionnaires online.

According to the principles of stratified proportional multi-stage cluster sampling, a total of 2300 individuals met the criteria for our survey. However, 243 respondents were excluded due to incomplete questionnaires or their inability to cooperate, resulting in a total of 2057 valid questionnaires with a pass rate of 89.4%. Among these, we collected 408, 350, 494, 276, and 529 questionnaires from Dongfang City, Haikou City, Wuzhishan City, Qionghai City, and Sanya City, respectively. The survey aimed to gather information on various gastrointestinal symptoms, such as diarrhea, abdominal pain, straining to defecate, sensation of incomplete evacuation, sensation of anal obstruction, belching, bloating, post-meal fullness, early satiety, burning sensation in the epigastric region, fecal character, and uncontrolled defecation, among others. We also inquired about the duration and frequency of these symptoms, previous medical experiences and examination results, general conditions (age, gender, place of residence, etc.), and factors that may be related to the disease, such as mental health, dietary habits, and sleep. Regarding medical experiences, we collected information on individuals' history of previous gastrointestinal diseases, other chronic illnesses, long-term medication use, as well as relevant examinations. Respondents who had undergone procedures such as gastroenteroscopy, abdominal ultrasound, fecal routine, occult blood test, or abdominal CT were asked to provide their results. These examinations helped in identifying organic gastrointestinal symptoms. Based on the questionnaire results, we advised individuals with suspected organic gastrointestinal pathology to undergo timely physical examinations or long-term follow-up. This approach helped in excluding patients who were suspected of having an organic disease at any time.

### Inclusion criteria and exclusion criteria

Inclusion criteria: Local residents of Hainan aged 18–85 years old who are willing to participate in the study and complete the questionnaire. Exclusion criteria: (1) Individuals suffering from severe mental illnesses that may hinder their ability to cooperate with the investigation^1^. (2) Individuals with diseases that could potentially impact the functioning of the digestive system, such as a history of abdominal surgery or digestive tumors. (3) Individuals presenting symptoms such as vomiting blood, black stools, recurrent mucus-pus-blood stools, and abdominal masses suggestive of possible digestive disorders. (4) Individuals on long-term use of analgesics, antiplatelet agents, antibiotics, or other medications that may have potential side effects. (5). Patients with epilepsy and Parkinson's disease, as these neurological disorders can cause gastrointestinal symptoms^48,49^.

### Diagnostic criteria and questionnaires

Baseing on the Rome IV Diagnostic Questionnaire for adults with FGID in conjunction with a validated Chinese version of the Rome IV questionnaire that have been applied^19,45,50^, on the basis of thorough investigation of the literature and consultation with experts, and taking into account the characteristics of China's Hainan Province, a questionnaire (preliminary draft) was designed to be applied to the study of this topic, and the final draft was finalised after the pre-survey was revised and improved. In addition, we have previously conducted a study of Post-infection FGID following coronavirus disease-19 using our adapted questionnaire, and the results have been published in international journals and cited by peers^19^.

### Definition

The study investigated several FGIDs, including FC, FDr, IBS, functional bloating, functional dyspepsia (FD), belching disorder, fecal incontinence, and functional heartburn, totaling eight disorders. The quality of sleep in patients was assessed using the Pittsburgh Sleep Quality Index (PSQI)^51^, where higher scores indicated poorer sleep quality. Poor sleep quality was defined as a score of more than 7, fair sleep quality as a score of 5–7, and good sleep quality as a score of less than 5. The psychological state of the case group was evaluated using the Hamilton Anxiety Scale^52^. Presence of anxiety was determined with a score of > 7, rare anxiety with a score of 5–7, and essentially no anxiety with a score of less than 5. The study also took into account previous diagnoses of psychiatric disorders, including depression, anxiety, obsessive–compulsive disorder, mania, etc. Smoking was defined as smoking every day or most days (> 5 days a week)^53^, while alcohol consumption was defined as drinking alcohol on a weekly basis for the past year^53^. Dietary irregularity was considered to occur when untimely or irregular meals happened frequently (> 5 times/month)^19^. In the Hainan region, people commonly consume betel nuts and pickled foods^38^. Consumption of pickled food and betel nut was defined as more than 3 times/week^19^. Examples of pickled foods include salted vegetables, salted fish, and dried radish. Betel nut, on the other hand, is the fruit of the betel nut tree, which grows in tropical areas^54^. Locals often chew betel nuts, usually wrapped in betel leaves and flavored with lime powder.

### Statistical methods

SPSS 20.0 statistical software was utilized to conduct data analysis. Continuous variables were presented as mean ± standard deviation (SD), and an independent samples *t*-test was employed for comparing groups. Categorical variables were expressed as frequency and percentage [n (%)], and the chi-square test was used to compare groups. All variables with a p-value of < 0.05 obtained from univariate analysis were included in the multifactorial logistic regression analysis, adopting a stepwise regression analysis approach with a SLS at 0.10 and SLE at 0.05. This analysis aimed to investigate potential relationships between FGIDs and risk factors. The results were reported as odds ratios (OR) with a 95% confidence interval (CI). Statistical significance was considered when the p-value was less than 0.05.

### Ethics approval and consent to participate

The protocol was approved by the institutional ethics committee of the Second Hospital of Hainan Medical University (LW2022126) and performed per Helsinki’s Declaration. All participants provided written informed consent for data collection and storage.

### Supplementary Information

Supplementary Figure S1.

Supplementary Figure S2.

Supplementary Table S1.

Supplementary Table S2.

Supplementary Table S3.

Supplementary Table S4.

Supplementary Table S5.

Supplementary Table S6.

## Data Availability

The datasets generated and/or analyzed during the current study are available from the corresponding author upon reasonable request.

## Abbreviations

FGIDs: Functional gastrointestinal diseases
FD: Functional dyspepsia
FC: Functional constipation
IBS: Irritable bowel syndrome
FDr: Functional diarrhea
PSQI: Pittsburgh Sleep Quality Index
