# Supplementary material for: Prevalence, types, and risk factors of functional gastrointestinal diseases in Hainan Province, China
Source: Sci Rep. 2024 Feb 24;14:4553. doi: 10.1038/s41598-024-55363-4 (PMC10894239; doi:10.1038/s41598-024-55363-4)
Supplement: Supplementary file 1 — Supplementary Figure S1. [file 41598_2024_55363_MOESM1_ESM.docx]

**Figure S1.** Urban distribution of major FGIDs in Hainan Province.
