# Supplementary material for: Prevalence, types, and risk factors of functional gastrointestinal diseases in Hainan Province, China
Source: Sci Rep. 2024 Feb 24;14:4553. doi: 10.1038/s41598-024-55363-4 (PMC10894239; doi:10.1038/s41598-024-55363-4)
Supplement: Supplementary file 3 — Supplementary Table S1. [file 41598_2024_55363_MOESM3_ESM.docx]

**Table S1: Univariate analysis of prevalence of functional dyspepsia**

| Indicator | Subgroup | Healthy group | Diseased group | X^2^ /t | P Value |
| --- | --- | --- | --- | --- | --- |
| Age(years) | 18-40 | 678 | 84 |  |  |
|  | 41-60 | 780 | 100 | 2.27 | 0.32 |
|  | >60 | 379 | 36 |  |  |
| Gender | Male | 520 | 66 | 0.28 | 0.59 |
|  | Female | 1317 | 154 |  |  |
| Sleep quality | Good | 647 | 60 | 6.07 | <0.05 |
|  | Average | 727 | 93 |  |  |
|  | Poor | 463 | 67 |  |  |
| Anxieties | Hardly | 877 | 91 |  |  |
|  | Occasionally | 647 | 85 | 3.34 | 0.19 |
|  | Often | 313 | 44 |  |  |
| Psychiatric disorders | No | 1789 | 213 | 0.24 | 0.62 |
|  | Yes | 48 | 7 |  |  |
| Educational level | Undergraduate and above | 498 | 59 |  |  |
|  | Elementary-High School | 1255 | 151 | 0.01 | 0.99 |
|  | Never attended school | 84 | 10 |  |  |
| Exercise duration/week | <1 hour | 863 | 111 |  |  |
|  | 2-4 hours | 558 | 71 | 3.30 | 0.19 |
|  | >4 hours | 416 | 38 |  |  |
| Smoking | not | 1546 | 167 | 9.60 | <0.05 |
|  | Yes | 291 | 53 |  |  |
| Drinking alcohol | not | 1610 | 173 | 13.80 | <0.05 |
|  | Yes | 227 | 47 |  |  |
| Eating pickled foods | not | 1294 | 149 | 0.69 | 0.41 |
|  | Yes | 543 | 71 |  |  |
| Edible betel nut | not | 1460 | 164 | 2.88 | 0.09 |
|  | Yes | 377 | 56 |  |  |
