# Supplementary material for: Prevalence, types, and risk factors of functional gastrointestinal diseases in Hainan Province, China
Source: Sci Rep. 2024 Feb 24;14:4553. doi: 10.1038/s41598-024-55363-4 (PMC10894239; doi:10.1038/s41598-024-55363-4)
Supplement: Supplementary file 4 — Supplementary Table S2. [file 41598_2024_55363_MOESM4_ESM.docx]

**Table S2: Multifactorial analysis of the prevalence of functional dyspepsia**

| Indicator | Subgroup | P Value | OR | 95% CI | |
| --- | --- | --- | --- | --- | --- |
|  |  |  |  | lowest | highest |
| Sleep quality | Good | 0.005 | 1.000 |  |  |
|  | Average | <0.05 | 1.505 | 1.096 | 2.066 |
|  | Poor | <0.05 | 1.728 | 1.231 | 2.427 |
| Smoking | not | <0.05 | 1.504 | 1.148 | 1.968 |
|  | Yes |  |  |  |  |
| Drinking alcohol | not | <0.05 | 1.682 | 1.228 | 2.304 |
|  | Yes |  |  |  |  |
