# Supplementary material for: Prevalence, types, and risk factors of functional gastrointestinal diseases in Hainan Province, China
Source: Sci Rep. 2024 Feb 24;14:4553. doi: 10.1038/s41598-024-55363-4 (PMC10894239; doi:10.1038/s41598-024-55363-4)
Supplement: Supplementary file 5 — Supplementary Table S3. [file 41598_2024_55363_MOESM5_ESM.docx]

**Table S3: Univariate analysis of prevalence of functional constipation**

| Indicator | Subgroup | Healthy group | Diseased group | X^2^ /t | P Value |
| --- | --- | --- | --- | --- | --- |
| Age(years) | 18-40 | 701 | 61 |  |  |
|  | 41-60 | 795 | 85 | 2.82 | 0.24 |
|  | >60 | 370 | 45 |  |  |
| Gender | Male | 534 | 52 | 0.17 | 0.69 |
|  | Female | 1332 | 139 |  |  |
| Sleep quality | Good | 656 | 51 |  |  |
|  | Average | 736 | 84 | 5.53 | 0.06 |
|  | Poor | 474 | 56 |  |  |
| Anxieties | Hardly | 880 | 88 |  |  |
|  | Occasionally | 667 | 65 | 0.97 | 0.62 |
|  | Often | 319 | 38 |  |  |
| Psychiatric disorders | No | 1815 | 187 | 0.27 | 0.60 |
|  | Yes | 51 | 4 |  |  |
| Educational level | Undergraduate and above | 513 | 44 |  |  |
|  | Elementary-High School | 1263 | 143 | 5.40 | 0.08 |
|  | Never attended school | 90 | 4 |  |  |
| Exercise duration/week | <1 hour | 868 | 106 |  |  |
|  | 2-4 hours | 585 | 44 | 6.90 | <0.05 |
|  | >4 hours | 413 | 41 |  |  |
| Smoking | not | 1557 | 156 | 0.39 | 0.534 |
|  | Yes | 309 | 35 |  |  |
| Drinking alcohol | not | 1615 | 168 | 0.29 | 0.59 |
|  | Yes | 251 | 23 |  |  |
| Eating pickled foods | not | 1321 | 122 | 3.96 | <0.05 |
|  | Yes | 545 | 69 |  |  |
| Edible betel nut | not | 1471 | 153 | 0.17 | 0.68 |
|  | Yes | 395 | 38 |  |  |
