# Supplementary material for: Prevalence, types, and risk factors of functional gastrointestinal diseases in Hainan Province, China
Source: Sci Rep. 2024 Feb 24;14:4553. doi: 10.1038/s41598-024-55363-4 (PMC10894239; doi:10.1038/s41598-024-55363-4)
Supplement: Supplementary file 6 — Supplementary Table S4. [file 41598_2024_55363_MOESM6_ESM.docx]

**Table S4: Multifactorial analysis of the prevalence of functional constipation**

| Indicator | Subgroup | P Value | OR | 95% CI | |
| --- | --- | --- | --- | --- | --- |
|  |  |  |  | lowest | highest |
| Exercise duration/week | <1 hour | 0.008 | 1.000 |  |  |
|  | 2-4 hours | <0.05 | 0.601 | 0.435 | 0.830 |
|  | >4 hours | 0.616 | 0.923 | 0.674 | 1.264 |
| Eating pickled foods | not | <0.05 | 1.357 | 1.049 | 1.755 |
|  | Yes |  |  |  |  |
