# Supplementary material for: Prevalence, types, and risk factors of functional gastrointestinal diseases in Hainan Province, China
Source: Sci Rep. 2024 Feb 24;14:4553. doi: 10.1038/s41598-024-55363-4 (PMC10894239; doi:10.1038/s41598-024-55363-4)
Supplement: Supplementary file 7 — Supplementary Table S5. [file 41598_2024_55363_MOESM7_ESM.docx]

**Table S5: Univariate analysis of prevalence of irritable bowel syndrome**

| Indicator | Subgroup | Healthy group | Diseased group | X^2^ /t | P Value |
| --- | --- | --- | --- | --- | --- |
| Age(years) | 18-40 | 724 | 38 |  |  |
|  | 41-60 | 815 | 65 | 7.36 | <0.05 |
|  | >60 | 378 | 37 |  |  |
| Gender | Male | 555 | 31 | 2.97 | 0.09 |
|  | Female | 1362 | 109 |  |  |
| Sleep quality | Good | 673 | 34 |  |  |
|  | Average | 765 | 55 | 11.09 | <0.05 |
|  | Poor | 479 | 51 |  |  |
| Anxieties | Hardly | 921 | 47 |  |  |
|  | Occasionally | 685 | 47 | 26.78 | <0.05 |
|  | Often | 311 | 46 |  |  |
| Psychiatric disorders | No | 1865 | 137 | 0.16 | 0.69 |
|  | Yes | 52 | 3 |  |  |
| Educational level | Undergraduate and above | 526 | 31 |  |  |
|  | Elementary-High School | 1305 | 101 | 2.09 | 0.35 |
|  | Never attended school | 86 | 8 |  |  |
| Exercise duration/week | <1 hour | 904 | 70 |  |  |
|  | 2-4 hours | 597 | 32 | 4.903 | 0.09 |
|  | >4 hours | 416 | 38 |  |  |
| Smoking | not | 1596 | 117 | 0.01 | 0.92 |
|  | Yes | 321 | 23 |  |  |
| Drinking alcohol | not | 1669 | 114 | 3.59 | 0.06 |
|  | Yes | 248 | 26 |  |  |
| Eating pickled foods | not | 1349 | 94 | 0.65 | 0.42 |
|  | Yes | 568 | 46 |  |  |
| Edible betel nut | not | 1507 | 117 | 1.93 | 0.17 |
|  | Yes | 410 | 23 |  |  |
