# Supplementary material for: Prevalence, types, and risk factors of functional gastrointestinal diseases in Hainan Province, China
Source: Sci Rep. 2024 Feb 24;14:4553. doi: 10.1038/s41598-024-55363-4 (PMC10894239; doi:10.1038/s41598-024-55363-4)
Supplement: Supplementary file 8 — Supplementary Table S6. [file 41598_2024_55363_MOESM8_ESM.docx]

**Table S6: Multifactorial analysis of prevalence of irritable bowel syndrome**

| Indicator | Subgroup | P Value | OR | 95% CI | |
| --- | --- | --- | --- | --- | --- |
|  |  |  |  | lowest | highest |
| Age(years) | 18-40 | <0.05 | 1.000 |  |  |
|  | 41-60 | 0.115 | 1.403 | 0.921 | 2.138 |
|  | >60 | <0.05 | 1.931 | 1.184 | 3.149 |
| Sleep quality | Good | 0.144 | 1.000 |  |  |
|  | Average | 0.225 | 1.321 | 0.842 | 2.071 |
|  | Poor | <0.05 | 1.598 | 1.002 | 2.549 |
| Anxieties | Hardly | <0.05 | 1.000 |  |  |
|  | Occasionally | 0.115 | 1.413 | 0.919 | 2.174 |
|  | Often | <0.05 | 2.801 | 1.796 | 4.366 |
